# A review of the bioelectronic implications of stimulation of the peripheral nervous system for chronic pain conditions

**DOI:** 10.1186/s42234-020-00045-5

**Published:** 2020-04-24

**Authors:** Timothy R. Deer, Ramana Naidu, Natalie Strand, Dawn Sparks, Alaa Abd-Elsayed, Hemant Kalia, Jennifer M. Hah, Pankaj Mehta, Dawood Sayed, Amitabh Gulati

**Affiliations:** Spine and Nerve Center for the Virginias, 400 Court Street, Suite 100, Charleston, West Virginia 25301 USA

**Keywords:** Peripheral nerve, Neurostimulation, Review, Chronic pain, Ultrasound guidance

## Abstract

**Background:**

Peripheral Nerve Stimulation has been used to treat human disease including pain for several decades. Innovation has made it a more viable option for treatment of common chronic pain processes, and interest in the therapy is increasing.

**Main body:**

While clinical data is forthcoming, understanding factors that influence successful outcomes in the use of PNS still needs to be delineated. This article reviews the evolution and bioelectronic principles of peripheral nerve stimulation including patient selection, nerve targets, techniques and guidance of target delivery. We collate the current evidence for outcomes and provide recommendations for salient topics in PNS.

**Conclusion:**

Peripheral nerve stimulation has evolved from a surgically invasive procedure to a minimally invasive technique that can be used early in the treatment of peripheral nerve pain. This review identifies and addresses many of the variables which influence the success of PNS in the clinical setting.

## Introduction

The peripheral nervous system is an integral part of the body’s communication with the environment (Kiernan et al., 2013). Touch, proprioception, temperature, and nociception influence our perception of the world (Benarroch et al., 2018). In most situations after mechanical or metabolic trauma, transduction and nociception can be beneficial, informing an organism to retreat or protect. However, persistence in nociception or peripheral nerve dysfunction can lead to the development of chronic pain which can have profound consequences to that organism and its social structure (Campbell, 2008; Costigan et al., 2009). Peripheral changes in chemical mediators can lead to pathological nerve firing, triggering changes in the cell bodies of somatosensory neurons located in the peripheral ganglia (dorsal root or trigeminal ganglia). These cell bodies serve as first pass junctions for the transmitted signal, and may lead to a hyperexcitable state with resultant changes in the area of the peripheral nerve, the peripheral ganglia, the spinal cord, and at the level of the anterior cingulate cortex (Tajerian et al., 2013). Common examples of chronic pain caused by peripheral nerve injury include ilioinguinal and/or iliohypogastric nerve pain after inguinal herniorrhaphy, sural nerve injury after podiatric surgery leading to foot pain, intercostal nerve pain after thoracotomy, and facial pain after ophthalmic infection of herpes zoster (Alfieri et al., 2006; Gerner, 2008; Primadi et al., 2016; Opstelten & Zaal, 2005).

Once chronic pain has developed, the patient may experience allodynia, hyperalgesia, and loss of movement or function (Bennett & Xie, 1988). These problems can lead to suffering and disability, which is a major economic burden to both the patient and the healthcare community (Breivik et al., 2006; Duenas et al., 2016). It is estimated that up to 10 % of those impacted by chronic pain may have an origin related to peripheral nerve pathology (Breivik et al., 2006). The diagnosis is made by pain in the distribution of the peripheral nerve, based on both history and examination. Imaging, such as MRI or ultrasound along with diagnostic tests, such as electromyelogram or nerve conduction studies are often used to aid in diagnosis (Rangavajla et al., 2014; Aminoff, 2004). The interventional pain physician may also do diagnostic peripheral nerve blocks using local anesthetic(s) to aid in diagnosis (Bates et al., 2019).

Treatment options for peripheral nerve injury include: physical therapy, oral medications, ablative therapies (e.g. thermal or chemical) and neurostimulation (Bates et al., 2019). If patients do not significantly improve with conservative therapies, PNS is a viable nerve treatment option to treat pain secondary to peripheral nerve injury (Van Buyten et al., 2015; Nagel et al., 2014; Pereira & Aziz, 2014). However, for PNS to be successful, understanding of outcomes is not sufficient. Thus, in this review we discuss the principles that govern successful outcomes for PNS. These principles include understanding the usefulness of a trial prior to an implant, optimal stimulation parameters, appropriate image guidance for optimal lead placement, and specific neural targets which have evidence for success. PNS is considered a low risk procedure, but also has a lower threshold for efficacy, based on limited high evidence studies (Deer et al., 2016).

### Historical context

PNS was first used in Philadelphia, Pennsylvania 60 years ago to treat pain in the head and neck (Shelden, 1966). The original approach described by Wall and Sweet was an open approach where the surgeon dissected the tissue to visualize the nerve and apply the lead (Long, 1977). This approach was modified and involved placing a transposed fascial graft between the nerve and lead, though fell out of favor due to the complexity of the surgery and adverse effects (Law et al., 1980). This procedure fundamentally changed in 1999, when Weiner described using percutaneous leads originally designed for the spinal cord to treat occipital nerve-induced headaches (Weiner & Reed, 1999). Eventually, a randomized multicenter prospective study was performed to access efficacy and safety of PNS for the migraine indication (Saper et al., 2011). Unfortunately, this potential landmark study failed to meet the primary end point and brought attention to major adverse events such as lead erosion and lead migration (Saper et al., 2011).

The first clinical study to discuss a device strictly designed to stimulate a peripheral nerve was published by Deer and colleagues as an Investigational Device Exemption (IDE) study on peripheral nerve pain (Deer et al., 2016). This device, the Stimrouter (Bioness, Valencia, California), obtained Food and Drug Administration (FDA) approval for PNS in the trunk and limbs. The differentiating feature was a small implantable tined lead, with a pickup contact that would be used with an external peripheral nerve generator (Pereira & Aziz, 2014). In the past decade, additional innovation has led to miniaturization of technology, education, improvement in placement based on imaging, and additional studies on efficacy and safety (Mansfield & Desai, 2020; Tubbs et al., 2015; Shaw et al., 2016).

## Potential mechanisms of peripheral nerve stimulation

PNS is the application of an electric field to a nerve or group of nerves including and/or distal to the dorsal root or trigeminal ganglion (Abejon & Perez-Cajaraville, 2011). Known as the gate control theory, peripheral nerve fibers, including A-alpha, A-beta, A-gamma, A-delta, various B and C fibers, are modulated when an electric field is applied (Melzack & Wall, 1965). The theory suggests that the paresthesia is elicited by an activation of the A-beta fibers which in turn activate the inhibitory interneurons and inhibit the C fibers from carrying afferent nociceptive input. In 1984, Chung et al., provided basic science research using a primate model substantiating this theory (Chung et al., 1984). This afferent inhibition has been the theoretical foundation in peripheral nerve stimulation.

In 2004, additional work by Chae et al. demonstrated that positive efferent stimulation could have an impact on pain perception in human subjects, improving pain and function (Chae et al., 2005; Qiu et al., 2019). This work also supports that PNS may affect the local concentration of biological mediators in the peripheral nervous system target. Chronic pain arising from the peripheral nerve increases the local concentration of mediators such as endorphins and prostaglandins, which leads to increases in blood flow. PNS may have a direct effect on reducing this increased concentration of bio-inflammatory mediators, blood flow and pain transmission (Papuc & Rejdak, 2013).

In addition to the peripheral mechanisms discussed here, there are additional theories suggesting there may be spinal or central neural pathways that lead to changes in the pain pathways (Vartiainen et al., 2009; Flor, 2002). Further human studies are needed to authenticate the potential mechanisms. fMRI research in animal models has revealed that the prefrontal cortex and limbic system change in their metabolism and scale after stimulation of peripheral nerves (Long, 1977; Weiner & Reed, 1999; Saper et al., 2011).

Electrical programming variables, such as choice in frequency, pulse width and intensity, have evolved over time. In general, low frequency tonic stimulation to activate either motor or sensory fibers, has been the standard for PNS waveform selection. Recently, higher frequencies (1 to 10 KHz) and novel waveforms, burst therapy, have influenced the spinal cord in unique ways (Amirdelfan et al., 2018; North et al., 2016; Manning et al., 2019). Novel high frequencies have been applied to PNS resulting in a blockade of nerve conduction using 10Khz frequency (Gilmore et al., 2019b).

While significant animal and computer modelling work has been done in the field of PNS, the bioelectronic principles to govern PNS waveform choices clinically are in its infancy. Once implanted in a patient, PNS systems are typically used to stimulate sensory nerve fibers, such as the tibial nerve. Interestingly, the use of PNS on the tibial nerve may result in treatments for sensory pain relief of the pelvis and foot, motor dysfunction, and in some settings for bladder dysfunction (van Balken et al., 2003; de Wall & Heesakkers, 2017). These results are not fully understood. Furthermore, optimal parameters to maximize the potential of PNS is still being defined in the clinical setting. Stimulation effects on a nerve are impacted by distance from the stimulator to the nerve, the impedance of the tissue, and the quality and consistency of the current delivery. Lead shape, consistency of the signal, and electrical field produced by the PNS system may result in unique and different effect on nerves, even if stimulation parameters are similar (Frahm et al., 2016; Morch et al., 2014). In the end, these bioelectronic effects on nerves are only beginning to be elucidated.

## Targets for peripheral nerve stimulation

### Neural targets for headaches and facial pain

The greater and lesser occipital nerves, which are branches of the C2 and C3 nerve roots, have long been a target for treatment of certain headache disorders, chronic post-traumatic pain and occipital neuralgia (Slavin et al., 2019). The occipital nerves innervate the skin overlying the occiput, and have their axons originating in the trigeminocervical complex. Stimulation of these nerves may result in treatment of headache disorders (Bartsch & Goadsby, 2003). Stimulation of the greater, the lesser, and the third occipital nerve with lead placement above and/or below the nuchal line may potentially stimulate one or all three of the occipital nerves (Hayek et al., 2009).

Similarly, supraorbital and infraorbital nerves may be targeted with stimulation techniques (Antony et al., 2019). The supraorbital nerve has a lateral branch, which provides sensory innervation to the skin of the forehead, and a medial branch, which innervates the nose, medial part of the upper eyelid, and medial forehead (Deer et al., 2016; Chung et al., 1984; Chae et al., 2005; Qiu et al., 2019; Papuc & Rejdak, 2013). The infraorbital nerve is a terminal branch of the maxillary nerve and has a sizable area of innervation. This includes sensation to the lower eyelid, the lateral nose, and the upper lip. Current literature supporting the use PNS for these nerves is limited to case series and low level evidence (Antony et al., 2019).

### Targets for neuropathies of the upper extremities

Mono-neuropathic pain syndromes of the upper extremity are potential targets for PNS. Common causes of nerve pathology include trauma and post-surgical nerve compression. The ulnar, median, and radial nerve can all be targets for PNS, but it may be technically difficult to place PNS leads due to proximity to the elbow and wrist (Arias-Buria et al., 2019). Ultrasound guidance is particularly helpful in mapping out lead placement and power source placement for the distal upper extremity (Huntoon et al., 2008).

The presence of shoulder pain is common in clinical practice and occurs from a variety of pathologies including osteoarthritis, trauma, post-surgical pain, and post-stroke pain. The axillary nerve may be stimulated to improve lateral shoulder pain and motor functional abnormalities, while the suprascapular nerve may be stimulated to improve glenohumeral joint pain and motor pathology of the supra and infraspinatus muscles. The suprascapular nerve can be modulated in the suprascapular notch above the scapular spine to target pain arising from the shoulder joint or from below the notch coming in from a lateral to medial approach (Gofeld & Agur, 2018). Ultrasound guidance has typically been used to identify the suprascapular notch and visualize, and thereby stimulate, the suprascapular nerve and artery, with evidence of efficacy the post-surgical setting. Fluoroscopic guidance may assist in optimal stimulator placement (Kurt et al., 2016). Similarly, the axillary nerve can be modulated by placing a PNS electrode near the nerve either at the quadrangular space or at the surgical neck of the where the nerve and circumflex humeral artery are visualized (Wilson et al., 2018). Research in shoulder pain is a topic of critical interest, and data is improving for both post stroke and degenerative shoulder disease (Mansfield & Desai, 2020).

### Neural targets for lower extremity neuralgias

When targeted either individually or in conjunction, neuromodulation of the femoral and sciatic nerves has resulted in significant improvement in patients suffering from phantom and residual limb pain after amputation (Gilmore et al., 2019a). PNS of the femoral nerve has resulted in treatment of post-operative pain relief after knee surgery (Ilfeld et al., 2019). Targeting the sciatic nerve independently for phantom limb pain has been an established therapy (Rauck et al., 2014). An interesting finding from Rauck et al. was determining the optimal distance the stimulating device is from the sciatic nerve. Optimal stimulation may occur 1 to 1.5 cm away from the sciatic nerve, which contradicts earlier techniques which place the stimulating leads next to a nerve.

Some interesting targets have emerged from work in this field. Finch et al. describe various lower extremity targets, including the sacral S1 nerve root using a novel 10 KHz waveform for pain relief (Finch et al., 2019). The tibial nerve has become a unique target because it is part of the craniosacral autonomic parasympathetic nervous system, and has both motor and sensory function. Lead placement is typically parallel to the nerve with the pulse generator or battery source on the calf. Current evidence may support PNS of the tibial nerve as a treatment option of overactive bladder (de Wall & Heesakkers, 2017; Staskin et al., 2012). A similar case may be made for stimulation of the saphenous nerve. Initial reports for the saphenous nerve target for overactive bladder may make PNS a useful modality in the future (MacDiarmid et al., 2018).

### Novel neural targets targeting the abdominopelvic wall

Common peripheral nerve targets for chronic inguinal or groin pain, the ilio-inguinal, genito-femoral and ilio-hypogastric nerves, are easily localized with ultrasound guidance and may be potential targets for peripheral nerve stimulation (Tubbs et al., 2015). Post-operative pain is the most common cause of inguinal, iliohypogastric and genitofemoral nerve pain, usually resulting from herniorrhaphy, hysterectomy, or iliac bone graft (Bouche, 2013). Surgical implantation of PNS systems has resulted in improvement for patients suffering from these ailments (Shaw et al., 2016). Whereas surgery may be a possible technique to identify these set of nerves, ultrasound guided procedures may be preferable to both physicians and patients. Elahi et al. describe a technique placing 2 eight contact electrodes in eight patients resulting in significant pain relief in a small cohort of patients (Elahi et al., 2015).

## Guidance methods for lead placement

Guidance methods for lead placement have changed with the evolution of PNS over time. As mentioned previously, direct cutdown to the nerve was used in the 1960s to facilitate placement of a paddle or percutaneous lead in close proximity to the target nerve under direct visualization (Costigan et al., 2009). Open lead implantation had several disadvantages including significant postoperative pain and scar tissue buildup (Costigan et al., 2009).

Guidance for peripheral nerve blockade has evolved providing several modalities to facilitate minimally invasive percutaneous approaches to lead placement for PNS. The PNS lead, consisting of a single or multiple electrodes, is implanted in close proximity to a target peripheral nerve in the perifascial plane without the need for a large surgical incision (Costigan et al., 2009; Saper et al., 2011). The battery source used to stimulate the lead is either implanted or remains external (Costigan et al., 2009; Tajerian et al., 2013). Confirmation of optimal peripheral nerve localization should occur through nerve stimulation with a goal of having the lead be parallel to the nerve (Breivik et al., 2006).

Traditional landmark-based techniques fail to account for anatomic variation which frequently occurs in the course of peripheral nerves. This is of particular concern in individuals with peripheral nerve injury resulting from an operation. As a result, normal anatomic landmarks may no longer indicate the location of peripheral nerves. Nonetheless, anatomic landmarks can facilitate peripheral nerve localization by providing a starting point for further identification. In the case of chronic neuropathic post-amputation pain, the femoral nerve is typically identified 1 to 2 cm distal to the inguinal crease and the sciatic nerve is typically identified by palpating the greater trochanter and ischial tuberosity as landmarks (Breivik et al., 2006). When lead placement requires superficial placement, as in the case of craniofacial stimulation, the use of external landmarks facilitates lead placement.

The use of fluoroscopic guidance for PNS lead placement has been described for a number of targets (Long, 1977; Deer et al., 2014). Fluoroscopic guidance allows for visualization of bony landmarks in the vicinity of the target nerve. The PNS lead can be advanced under intermittent visualization and adjusted as needed with clear documentation of final placement. Compared to ultrasound guidance, fluoroscopy does not allow for visualization of vascular structures or the nerve target itself. Further research is needed to determine the comparative effectiveness and safety of the various image-guided techniques.

Imaging modalities are often combined during PNS lead placement (Weiner & Reed, 1999). For instance, fluoroscopic guidance can be used to initially identify a target lumbar level for stimulation of a lumbar medial branch, with subsequent use of ultrasound guidance for PNS lead placement. Then, fluoroscopy can be used for confirmation of final lead placement at the target lumbar level. A similar sequence of imaging modalities can also be used to identify a target intercostal space for stimulation of a given intercostal nerve via fluoroscopy, with PNS lead placement via ultrasound guidance, and confirmation of final lead placement at the target intercostal space via fluoroscopy (Weiner & Reed, 1999).

An example of using only fluoroscopy for PNS lead placement is seen in the head and neck patient population. When placing a lead for trigeminal branch stimulation of the supraorbital or infraorbital nerves, a Touhy needle can be advanced subcutaneously under intermittent fluoroscopic guidance to the supra- or infra- orbital regions either 1 cm above or below the orbital rim until the distal aspect of the lead reaches the medial border of the orbit (Costigan et al., 2009). Fluoroscopic guidance has been similarly described for placement of occipital leads (Weiner & Reed, 1999). The approach is described as a horizontal placement across the greater, lesser, and the third occipital nerves with tunneling towards the parieto-occipital region (Chung et al., 1984). Compared to fluoroscopy alone, the combined use of fluoroscopy and ultrasound guidance for occipital nerve stimulator implant has not been associated with increased lead survival (Deer et al., 2014).

Nerve stimulation remains a cornerstone for lead placement throughout the implantation process alone or in combination with other guidance methods (Breivik et al., 2006; Saper et al., 2011). Most commonly, ultrasound or fluoroscopic guidance is used for initial target localization, with use of nerve stimulation for confirmation. Thus, nerve stimulation assures identification of the correct peripheral nerve target prior to final implantation. Throughout lead placement, the amplitude needed to produce a paresthesia in the sensory distribution of the target nerve signals proximity to the nerve target. Thus, a higher mA output needed to produce a paresthesia indicates the stimulation probe or PNS lead is further from the intended target (Deer et al., 2016). Specifically, PNS should be targeted to the area where nerve connections are still intact, typically corresponding to the hyperalgesia in the region around the area of allodynia (Costigan et al., 2009; Long, 1977). Targeting paresthesia coverage over the area of allodynia may lead to failure of PNS due to targeting of damaged nerve tissue (Long, 1977).

Nerve stimulation may serve an important role to mimic the outcome of PNS lead placement during the implantation process. Based on the response to nerve stimulation, lead placement can be adjusted incrementally to optimize lead placement. During lead placement for post-amputation pain, the desired response to nerve stimulation is the production of comfortable paresthesia in the amputated foot or leg with minimal subcutaneous sensations proximal to the lead in the skin over the upper thigh or buttock (Deer et al., 2016). This corresponds to PNS of the nerve rather than the activation of subcutaneous afferents superficial to the electrode. By minimizing incisions around the proximal receiver end during percutaneous PNS lead implantation coupled with an external pulse transmitter (Chung et al., 1984), it is now possible to provide PNS in the recovery room immediately after implantation.

Aside from the use of nerve stimulation to produce a sensory paresthesia, PNS lead placement can also be confirmed with corresponding motor stimulation. For example, to target the medial branches of the dorsal rami nerves under ultrasound guidance, nerve stimulation with selective activation of the lumbar multifidus muscles with corresponding comfortable contractions overlapping the painful region have been used to guide PNS lead implantation (Gilmore et al., 2019b). Nerve stimulation has been described as an important technique for lead placement to treat hemiplegic shoulder pain. Monopolar needle electrodes are inserted perpendicular to the skin to localize the axillary nerve along the middle and posterior deltoids. A point between these locations is identified with adjustment of needle position and depth until both heads of the deltoids contract with full reduction of subluxation. The PNS lead is then inserted to the location indicated by the monopolar needle electrode (Chae et al., 2005).

One of the greatest advancements in the development of peripheral nerve stimulation over the past two decades has been the improvement in quality and cost reductions in ultrasound-imaging technology. This technology, carried over from the concepts of regional anesthesia, has provided the ability for a clinician to visualize a nerve and the adjacent structures to avoid, all in real time (Marhofer & Chan, 2007). Furthermore, ultrasound machines are increasingly portable allowing providers to carry them to any location. The use and education of ultrasound has increased significantly in anesthesiology, particularly regional anesthesia, and neuromodulation (Melnyk et al., 2018). In a more widespread fashion, medical education continues to emphasize the use of ultrasound in cadaveric teaching and the instrument is becoming as accepted to the modern-day physician as the stethoscope (Hoppmann et al., 2011).

Understanding of visual and tactile anatomy is still extremely important in ultrasound-guidance. It provides a place to initiate imaging. Sonographic anatomy is dependent on understanding relationships among anatomic structures. These relationships can help “triangulate” certain structures that may appear different in varying individuals (Ihnatsenka & Boezaart, 2010). Peripheral nerves often travel adjacent to arterial structures. Therefore, the use of doppler or color modes can help identify vascular structures that queue in on a nerve target. Also, these vascular structures should be avoided so that proper electric field can provide benefit, and so there is no increased bleeding from the procedure itself (Chan et al., 2010).

During needle entry, visualization can be optimized with echogenic needles and or with beam-steering to enhance the visualization of the needle (Hebard & Hocking, 2011; Prabhakar et al., 2018). The needle can be positioned near the nerve in an orthogonal trajectory or in parallel. Although distance is determined by which system the operator chooses, it is important to avoid mechanically damaging the nerve, which can be visualized with ultrasound imaging when in-plane. Depending on the peripheral nerve stimulator system, it is generally desired to place a lead parallel along the nerve so that if migration does occur, there is less risk of losing therapeutic benefit (Frahm et al., 2016). Another advantage of ultrasound guidance is real-time visualization during hydro dissection to avoid damage to neural and vascular structures in close proximity to the PNS target. This technique has been described to facilitate percutaneous implantation of PNS leads close to the suprascapular nerve or the cervical nerve roots within the brachial plexus to treat chronic neuropathic pain of the upper extremity (Tajerian et al., 2013).

## To trial or not to trial

Unlike spinal cord stimulation, a consistent technique for trialing PNS has not been developed. Part of this is dependent on current technology constraints, costs, and partly due to our limited understanding of the long-term mechanisms of action which may determine efficacy of nerve stimulation (Chakravarthy et al., 2019). This section will review current recommendations and thoughts on potential benefits of trialing techniques.

One common strategy is to employ a local anesthetic block of a peripheral nerve which may blunt the transmission of the pain signal (Chung & Spangehl, 2018). The rationale in this theory is that a conduction block will replicate PNS, however this may be flawed since current cathode stimulating devices either activate motor or sensory fibers as opposed to block conduction (Pereira & Aziz, 2014). Thus, while a test block may alleviate pain, electrical stimulation may or may not result in the same response or vice versa (Sweet, 1976). It is important to think of the local anesthetic block as part of the diagnostic workup, but not a prognostic factor to whether neurostimulation will be successful.

Despite the lack of correlating evidence between the block and PNS some benefits of the local anesthetic block exist. First, the block may determine appropriate targets to apply electrical activity (Manning et al., 2019). This may include an understanding of anatomy surrounding the nerve, especially if ultrasound is used. Second, newer technologies which may utilize anodal blockade of neural current may replicate a local anesthetic blockade (Gilmore et al., 2019b). Finally, if a local anesthetic block did not alleviate pain, perhaps another target may be considered, with a caveat that electrical stimulation may still be effective regardless of results of the local anesthetic block. Local anesthetic block should be considered for patient confidence in an appropriate target, not necessarily for efficacy of PNS on the same neural target.

A simple strategy for trialing may be the application of TENS units near the area of perceived pain. Similar waveforms, motor or sensory frequency and pulse width, may be considered to replicate the waveforms of the PNS system. Unfortunately, direct stimulation of a peripheral nerve may distribute the signal in a different dermatomal pattern that is achievable by TENS. TENS trials have no definitive relationship correlation with success in spinal cord or peripheral nerve stimulation (Kirsch et al., 1975; Picaza et al., 1977; Schwarm et al., 2019). Thus, TENS trial is not routinely recommended for consideration of success of PNS systems.

Similarly, little evidence exists for the use of percutaneous electrical nerve stimulation (PENS) as a surrogate trial for PNS. While direct stimulation of a nerve may be possible, replicating the same waveform pattern is technically difficult. Furthermore, a short PENS treatment may not elicit the long term changes a PNS system may induce, leading to possible false negatives. While not described in the literature, the authors of this section have had negative experiences anecdotally with this trialing method, thus leading to PENS not being actively recommended as a trial for PNS.

Currently, spinal cord or dorsal column stimulators initially have a trial period where a patient gets to experience neurostimulation without a surgical cutdown. One advantage of this technique is one will be able to mimic the proprietary waveform for each technology in that individual’s daily life (Amirdelfan et al., 2018; North et al., 2016). Peripheral nerve stimulator trials can also be performed for 3–60 days, although long-term effects of PNS will not be determined. These trials can give the patient the experience of managing the device, as well as functional improvements, quality of life improvements and analgesia associated with the stimulation. Rauck et al. describe a two-week trial targeting the femoral or sciatic nerve for the use of PNS in the post-amputee population (Rauck et al., 2014). Dodick DW et al. describes a prescreening trial for a quadripolar lead for the treatment of migraines by targeting the occipital nerve (Dodick et al., 2015). The pre-screen eliminated 20 patients from a sample size of 177 patients who did not have 50% reduction in pain or paresthesia coverage thus reducing the cost of unnecessary implantation with explantation. Deer et al. described a PNS system that has the trial targeted on the day of the permanent implant informing us that different technologies warrant different considerations with regards to trialing (Deer et al., 2016).

## Evidence for the use of peripheral nerve stimulation

PNS utilization in named peripheral nerves produce consistently high success rates in achieving pain relief in well selected patients when delivered by skilled clinicians (Pereira & Aziz, 2014). Since the initial percutaneous devices were described there have been numerous case reports and case series published exemplifying the efficacy and safety of peripheral nerve stimulation (Chakravarthy et al., 2016; Manchikanti et al., 2014). The evidence grading (Table 1) can be used to examine the current state of the field. (Table 2). Ref Manchinkotti.).

**Table 1:** Qualified modified approach to grading of evidence

Level I	Strong	2 or more relevant high quality RCT”s for effectiveness, or 4 or more relevant high quality observational studies of large case series for assessment of preventive measures, adverse, consequences, and effectiveness of the other measures
Level II	Moderate	At least 1 relevant high quality RCT or multiple relevant moderate or low quality RCT”s or at least 2high quality relevant observational studies or large case series for assessment of preventive measures, adverse, consequences, and effectiveness of the other measures
Level III	Fair	At least 1 relevant high quality nonrandomized trial or observational study with multiple moderate or low quality observational studies, or at least on high quality relevant observational study or large case series for assessment of preventive measures, adverse, consequences, and effectiveness of the other measures
Level IV	Limited	Multiple moderate or low quality relevant observational studies, or moderate quality observational studies of large case series for assessment of preventive measures, adverse, consequences, and effectiveness of the other measures
Level V	Consensus Based	Opinion or consensus of large group of clinicians for effectiveness as well as to assess preventive measures, adverse, consequences, and effectiveness of the other measures

**Table 2:** Peripheral nerve stimulation: case series/reports

Study	Etiology	Study design	Nerves Targeted	Results	Conclusion
Huntoon & Burgher (2009)	Upper and Lower extremity neuropathic pain	Case series *N* = 8	Median Radial Ulnar Peroneal Post Tibial	43% patients reported > 80% improvement in their baseline pain	Level IV Minimally invasive ultrasound guided peripheral nerve stimulation provided effective pain control in patients with positive trial
Simopoulos et al. (2010)	Chronic Migraine	Case Report	Bilateral Auriculotemporal	NRS reduction from 9 to 5 in 16 month F/u MIDAS improvement from Grade IV to II	Level V Auriculotemporal nerve stimulation may be a promising therapy for patients with temporal migraine
Vaisman et al. (2012)	Intractable Trigeminal Autonomic Cephalgia	Case series *N* = 5	Supratrochlear Supraorbital	-VAS reduction from 8.9 to 1.6 − 3 patients reported complete wean off opioids	Level IV Supraorbital and supratrochlear stimulation is effective and safe as therapy for trigeminal autonomic cephalgia
Hann & Sharan, (2013)	Chronic Migraine	Case series *N* = 14	Occipital Supraorbital	71% reduction in severity and frequency of Headaches 50% resolution of headache symptoms	Level IV Dual Occipital nerve and supraorbital nerve stimulation may improve frequency of pain relief, neurological symptoms in chronic migraine
Reverberi et al. (2014)	Causalgia Neuropathic pain	Case series *N* = 15	Suprascapular Mandibular Occipital Brachial Plexus Ulnar Median Radial Intercostal Post Tibial Common Peroneal	After avg. 9.3 months follow up: - NRS score down to 3.46 - Opioid consumption reduced by 50%	Level IV Peripheral Nerve Stimulation is an effective modality in managing severe neuropathic pain following multiple joint surgeries that are complicated by causalgia.
Reed et al. (2015)	Hemiplegic Migraine	Case Series *N* = 4	Occipital Supraorbital	− 92% reduction of headache frequency − 44% reduction in VAS − 96% reduction in drug usage	Level IV Concordant combined occipital and supraorbital nerve stimulation was preferred by patients and suggest efficacy in treating both pain and motor aura in hemiplegic migraine
Oswald et al. (2019)	Mononeuropathies	Case Series *N* = 39	Axillary Genitofemoral Intercostal Ilioinguinal Lateral Femoral Cutaneous Peroneal Saphenous Suprascapular Sural Tibial	71% reduction in pain scores 72% improvement in activity	Level IV Peripheral nerve stimulators are new minimally invasive neuromodulation modality that shows promising early results
Stevanato et al. (2014)	Brachial Plexus Injury	Case Series *N* = 7	Brachial Plexus	Long term follow up at 6 months and 12 months reported 76.2 and 71.5% reduction in NRS scores respectively.	Level IV Surgical Implantation of quadripolar electrode leads provide long term successful pain relief in brachial plexus injuries
Wilson et al. (2014)	Subacromial Impingement Syndrome	Case series *N* = 10	Axillary Nerve	Significant reduction in pain compared to baseline	Level IV Peripheral Nerve stimulation is effective is treating chronic subacromial impingement syndrome

The occipital neurostimulation study, ONSTIM, was a prospective single-blind randomized study that enrolled 66 patients suffering from chronic migraine. The responder rate was 39% in the stimulation group and 6% in the preset arm. Success was defined as more than 50% reduction in their headache days or three point improvement in VAS (Saper et al., 2011).

Deer et al. published results of their prospective multicenter randomized double-blind partial crossover study, a total of 147 patients were consented and screened for the study, 3 months after randomization to treatment, active stimulation arm achieved a statistically significant higher response rate of 38% vs the 10% rate found in the control group (*p* = 0.0048). The treatment group specifically, reported mean pain reduction of 27.2% from baseline to 3 month follow-op compared to 2.3% reduction in control group (*p* < 0.0001). There were no adverse events reported (Deer et al., 2016; Deer et al., 2010; Deer et al., 2012; 510(k) Premarket Notification, 2019a). Rauck et al. performed a prospective open label feasibility study with 16 patients suffering from post-amputation pain syndrome. Targets were sciatic and femoral nerves statistically significant improvement was shown in quality of life and decrease in Beck Depression Inventory score (Rauck et al., 2014). A novel system was studied by Gilmore et al. prospectively for up to 60 days in the back and/or extremities for symptomatic relief of chronic intractable pain, postsurgical and posttraumatic acute pain (510(k) Premarket Notification, 2019b). The lead used had a novel structure that made 60 day trial possible with a reduction in infection risks.

Table 3 summarizes some of the seminal prospective studies in the field of peripheral nerve stimulation.

**Table 3:** Peripheral nerve stimulation: prospective studies

Study	Etiology	Study design	Outcome Measures	Results	Conclusion
Deer et al. (2010)	Upper Extremity mononeuropathy	Case series *N* = 10 Median nerve	VAS scores BPI	−20% of the patients reported 40–100% pain relief No adverse effects occurred	Level II Temporary implant resulted in both pain reduction and reduced use of oral opioid pain medication during the 5-day stimulation period.
Saper et al. ONSTIM Study (2011)	Chronic Intractable Migraine	RCT *N* = 66 Occipital Nerve	-Headache days -Pain intensity POMS MIDAS SF-36	Responder defined as > 50% reduction of headache days 39% responder rate in Adjustable stim arm 6% responder rate in preset stim arm	Level II Occipital nerve stimulation is effective in treating intractable Chronic Migraine
Rauck et al. (2014)	Post Amputation Pain	Prospective Open Label Feasibility Study *N* = 16 Sciatic and Femoral Nerves	-Brief Pain Inventory -Beck Depression Inventory	At the end of 4wks subjects reported improvement in their quality of life and decrease in Beck Depression Inventory score	Level III Peripheral nerve stimulation can be effective in achieving significant pain relief and improvements in quality of life in patients suffering with post amputation pain syndrome
Wilson et al. (2014)	Chronic Hemiplegic Shoulder pain	Prospective randomized control trial *N* = 25 Intramuscular Deltoid, axillary nerve	-Brief Pain -Inventory SF 3	At 16wks, the mean severity rating of BPI index decreased from 7.5 to 3.2 in the peripheral nerve stimulation arm	Level II Short term peripheral nerve stimulation is safe and efficacious treatment for shoulder pain.
Deer et al. (2016)	Chronic Neuropathic pain	Prospective multicenter randomized double-blind partial crossover study *N* = 94 Upper and lower extremity and trucal nerves	-NRS -BPI -Quality of life -Patient Satisfaction -Pain Medication	Avg pain reduction of 27% in the treatment group as compared to 2% in the control group	Level II Peripheral nerve stimulation is safe and effective treatment strategy to target neuropathic pain of peripheral nerve origin
Ilfeld et al. (2017)	Total Knee arthroplasty	Prospective open label feasibility study Femoral and Sciatic nerve	VAS	Reduction in VAS scores by an average of 63% at rest, with 4 of 5 subjects having relief of > 50%	Level III Ultrasound-guided percutaneous peripheral nerve stimulation may be a practical modality for the treatment of postsurgical pain

## Future directions of peripheral nerve stimulation

Recently, several PNS devices have come to market which are specifically designed to target a peripheral nerve (Pereira & Aziz, 2014; Tubbs et al., 2015). Historically, PNS was performed utilizing dorsal column spinal systems and bulky internal pulse generators (Antony et al., 2019). Newer systems using lower profile micro-leads, miniaturized internal pulse generators, and external pulse generators have decreased the invasiveness and improved safety and efficacy of peripheral nerve stimulation. As a result, outcomes for peripheral nerve stimulation by focusing on novel targets is an area of intense research and clinical focus (Finch et al., 2019). The future use of these devices are in line with the discussion of targets such as axial back stimulation, facial nerve stimulation, and novel non-pain indications such as posterior tibial nerve for incontinence, and hypoglossal nerve for sleep apnea (de Wall & Heesakkers, 2017; Deckers et al., 2018; StimRelieve, LLC, 2019; Eastwood et al., 2011). Of great interest is utilizing novel waveforms and frequencies which improved outcomes in spinal cord stimulation (Finch et al., 2019) and applying them to PNS*.* A pivotal study is underway evaluating high frequency stimulation of the sciatic nerve for phantom limb pain, based on a feasibility study by Soin et al. (Soin et al., 2015). Given the technological advancements, expanded indications, new targets, and recent improvements in reimbursement, PNS is well positioned to be an important part of future treatment strategies.

## Conclusion

The use of PNS has been an option for over 60 years for physicians offering neuromodulation to patients. Despite previous PNS therapies, we are currently entering a renaissance which is inspired by new devices engineered for specific peripheral use, improved guidance to place the leads, and an improvement in safety. The greatest need to further improve this biolelectronic therapy is in researching outcomes to help us better understand proper use and patient selection. This review identifies principles that a practitioner needs to address prior to implanting a PNS system in a patient. Best use of image guidance, selection of neural target, optimizing stimulation parameters, understanding trialing benefits and specific techniques are critical for the success of PNS outcomes in the clinical setting.

## Data Availability

References are listed in the references section as well as evidence tables in the manuscript.

## Abbreviations

DRG: Dorsal Root Ganglion
FDA: Food and Drug Administration
IDE: Investigational Device Exemption
PENS: Percutaneous Electrical Nerve Stimulation
PNS: Peripheral Nerve Stimulation
SCS: Spinal Cord Stimulation
TENS: Transcutaneous Electrical Nerve Stimulation
